# Development of a quality of work life scale for Japanese community pharmacists: a questionnaire survey mostly in large companies

**DOI:** 10.1186/s40780-024-00335-z

**Published:** 2024-03-11

**Authors:** Yuta Kato, Takashi Sekiya, Ryo Ishii, Yoji Hirako, Hiroki Satoh, Hiromichi Kimura

**Affiliations:** 1 Department of Institute for Future Initiatives, University of Tokyo, 7-1-3 Hongo, Bunkyo-Ku, Tokyo, 113-0033 Japan; 2 Graduate School of Pharmaceutical Sciences, University of Tokyo, 7-3-1 Hongo, Bunkyo-Ku, Tokyo, 113-0033 Japan; 3 Nippon Pharmacy Association, 3-12-2 Nihonbashi, Chuo-Ku, Tokyo, Japan

**Keywords:** Community pharmacists, Human resources, Job satisfaction, Quality of working life, Questionnaire, Survey

## Abstract

**Background:**

Human resource management may become complex for community pharmacists owing to recent changes in work volume and content. Few studies have examined job satisfaction, well-being, and quality of work life (QWL) among community pharmacists in Japan. This study focused on QWL, a more comprehensive concept than job satisfaction, and aimed to develop the QWL questionnaire for Japanese community pharmacists (the QWLQ for JCP) and assess its reliability and validity.

**Methods:**

A questionnaire survey was conducted among 2027 pharmacists who worked in pharmacies with the cooperation of 20 corporations running pharmacies. Collected data were subjected to principal component factor analysis with Promax rotation via SPSS Windows version 28.

**Results:**

The factor analysis used data from 1966 pharmacists. In total, five significant components, which formed the basis of the QWLQ for JCP, were identified. These included “Influence of work on mind and body,” “Relationships with colleagues,” “Relationship with the boss,” “Meaning of existence in the workplace,” and “Pride in work.” Cronbach’s alpha, which expressed reliability, ranged from 0.585 to 0.854 for all the subscales.

**Conclusion:**

The QWLQ for the JCP significantly explained the concept of QWL, which indicated that its validity was sufficient.

**Supplementary Information:**

The online version contains supplementary material available at 10.1186/s40780-024-00335-z.

## Background

Quality of work life (QWL), a multi-dimensional construct related to an individual’s personal and work life, has varying dimensions across organizations. In general, QWL can be conceptualized as a measure of job satisfaction and organizational commitment. Furthermore, its definition can also extend to an individual’s work and life experiences [1–4]. Morita noted the balance between personal and work life as an important variable in QWL [5]. However, researchers have varying theoretical perspectives, which lead to no consistent definition of QWL [6].

Lee [7] developed a QWL scale for nursing home staff in Japan based on Alderfer's ERG theory, which was a three-fold conceptualization of human needs: existence, relatedness, and growth. These QWL components included “Satisfaction with treatment,” “Satisfaction with relationship with boss,” “Satisfaction with relationships with colleagues,” and “Growth satisfaction.”

Nanjundeswaraswamy [8] developed a scale to measure nurses’ QWL. This scale comprised nine components: “work environment,” “working condition,” “work–life balance,” “compensation,” “relationship and cooperation,” “stress at work,” “job satisfaction,” “career development,” and “organizational culture.” Zaman and Ansari [9] used an exploratory factor analysis and developed a QWL scale for medical residents. It comprised five dimensions: “pay and benefits,” “supervision,” “intra-group relations,” “working conditions,” and “training.”

Some previous studies assessed QWL among pharmacists. McHugh [10], along with American Pharmaceutical Association (APhA) members, assessed “job satisfaction,” “career satisfaction,” “organizational commitment,” “turnover intention,” “likelihood of voting for a union,” and “patient care issues” as dimensions of QWL [10]. The Midwest Pharmacy Workforce Research Consortium conducted the 2014 National Pharmacist Workforce Survey to collect reliable information on the pharmacist workforce in the United States. It was the fourth consortium and continued the analyses and trends of previous surveys conducted in 2000, 2004, and 2009. The assessed QWL dimensions included: “stress control,” “job satisfaction,” “professional commitment,” “work-home conflict,” and “organizational commitment” [11]. Schommer identified pharmacists’ QWL components, such as “time stress,” “responsibility stress,” “level of control,” “work in harmony with home life,” “home life in harmony with work,” “job satisfaction,” “professional commitment,” and “organizational commitment” [12].

Ono [13] reviewed QWL research in the United States and identified 21 components: “bosses’ trust in subordinates,” “abilities of and respect for subordinates,” “diversity of work,” “work content that can be utilized in the future,” “self-respect,” “impact of non-work life on work life,” “degree to which work contributes to society,” “fair and adequate wages, and remuneration and its stability,” “institutional aspects of labor,” “legal equality at work,” “feedback of meaningful information and results,” “autonomy,” “participation and decision making,” “working conditions including physical conditions,” “social integration within the workplace,” “opportunities for growth and learning,” “meaningfulness of work,” “use of ability,” “job satisfaction with mental and physical health,” “employment stability and health,” and “responsibility” [13]. Most QWL components identified by Ono [13] were also included in other QWL studies. A literature review revealed that different instruments were used to measure QWL for different occupations and countries.

In Japan, the role of community pharmacists has recently expanded. In 2015, the Ministry of Health, Labour and Welfare (MHLW) declared its *Pharmacy Vision for Patients*, and proposed that community pharmacists shift from a drug-oriented to patient-oriented approach [14]. Community pharmacists were expected to comprehensively, continuously, and centrally manage their patients’ pharmaceutical care needs. Furthermore, they were required to provide patient-visiting services and 24-h consulting support among other services. Additionally, community pharmacists were expected to play a role in medication therapy management by collaborating with physicians and other healthcare providers. However, the Japanese healthcare system was traditionally physician-centered, and pharmacists' role focused on drug-oriented approaches, such as dispensing with accuracy. Hence, human resource management for community pharmacists may be difficult owing to recent and drastic changes in their work volume and content.

Human resource management, such as improving job satisfaction, is a valuable skill in the healthcare field as it influences the quality of care [15–17]. Studies reported that job satisfaction was positively associated with pharmaceutical service quality among community pharmacists [18, 19]. Furthermore, there was an equivocal relationship between workload, quality of care provided to patients [20–22], and pharmacists’ quality of work life (QWL) [23, 24].

However, in Japan, few studies have examined job satisfaction, well-being, and QWL among community pharmacists. Therefore, this study focused on QWL, a more comprehensive concept than job satisfaction, and aimed to develop a multi-trait-based QWL questionnaire for Japanese community pharmacists (the QWLQ for JCP) and psychometrically assess its reliability and validity.

## Methods

The QWLQ for JCP was developed in three phases: 1) development of a preliminary version of the QWLQ for JCP, 2) conduction of a pilot study to assess the preliminary version, and 3) field testing to determine and confirm the validity and reliability of the final version of the QWLQ for JCP with the target population.

### Phase 1: Development of the preliminary version of the QWLQ for JCP

A literature review was conducted to understand the multiple dimensions of QWL experienced by community pharmacists. We used Ono’s 21 QWL components [13], and developed a preliminary version of the QWLQ for JCP. The items were also developed with reference to several previous studies on QWL or job satisfaction [25, 26]. The preliminary version was confirmed and revised by community pharmacists for comprehensive coverage. The preliminary version included 63 items (three items per component) and was administered to Japanese pharmacists.

To confirm the scale’s validity and reliability, we used the 15-item QWL scale developed by Lee [7] and a three-item QWL questionnaire used by an older adult welfare facility staff in Japan. These items focused on “overall job satisfaction,” “intention to continue working,” and “life satisfaction other than work” [7].

Responses were rated on a 6-point Likert scale that ranged from (1) I strongly disagree, (2) I mostly disagree, (3) I disagree a little, (4) I agree, (5) I mostly agree, to (6) I strongly agree. We calculated each QWL score, and higher scores indicated higher levels of QWL satisfaction.

Furthermore, seven items were used to obtain participants’ information regarding “gender,” “age,” “employment status,” “length of service in a company,” “years of pharmacist experience,” “working hours per week,” and “average number of prescriptions filled per day.”

### Phase 2: Pilot study to assess the preliminary version of the QWLQ for the JCP

A pilot study was conducted to evaluate the content and validity of the preliminary version of the QWLQ for JCP. We requested pharmacy companies to administer the questionnaires to their pharmacists. We explained that this survey was anonymous and that the companies could not see the completed questionnaire to encourage voluntarily participation.Participants worked as pharmacists in community pharmacies owned by a company in the Kanto area of Japan. Each received the questionnaire from headquarters via e-mail and returned the completed questionnaire to the headquarters via email. We received the completed questionnaires from the company. The questionnaire survey was anonymous, and we ensured that the participants agreed to participate by completing the questionnaire.

We confirmed that each item was appropriate for the preliminary version of the QWLQ for JCP. First, for item analysis, responses were evaluated for completeness of data and variance in item responses. We confirmed the absence of ceiling and floor effects. In addition, since we aimed to develop a scale to measure individual differences in QWL, we considered that items with little response variance were not appropriate. Therefore, items where 50% of the respondents selected the same option were excluded. Next, we calculated correlation coefficients to confirm whether the items had similar content. For multiple items with high correlation coefficients and similar content, one item with lower variance was excluded. This was based on the theory that a measurement scale should not contain multiple items that described the same question [27].

Second, we calculated regression coefficients to confirm whether the preliminary version of the QWLQ for JCP explained the QWL scale (15 items) developed by Lee [7] and QWL questionnaire (three items). Through this analysis, we developed a pre-final version of the QWLQ for JCP.

Finally, we conducted a principal component factor analysis with Promax rotation to compare the results of the pilot study with those of the field study.

### Phase 3: Field study to develop the QWLQ for the JCP

#### Participants and data collection

We requested the headquarters of each pharmacy company that agreed to participate to distribute the questionnaire to community pharmacists via e-mail between May and June 2022. The participants were anonymous volunteers. To encourage voluntariness in participation, participants were informed that the participation was voluntary and that their responses would not be seen by the company. After each participant received a questionnaire, they read the explanation document. If they agreed to participate in the survey, they returned the completed questionnaire directly to our researchers via Microsoft Form system.

The questionnaire included the pre-final version of the QWLQ for the JCP (43 items), QWL scale (15 items) developed by Lee [7], QWL questionnaire (three items), and participants’ background questionnaire (eight items).

#### Methods of analyses

To verify the validity and reliability of the final version, we performed item and factor analyses. For item analysis, the responses were evaluated similar to that in the pilot study (see Phase 2).

To determine the internal factor structure, exploratory factor analysis was performed on a new set of items after item analysis. Principal component factor analysis with Promax rotation was applied to the exploratory factor analysis. Factors with eigenvalues greater than 1 were retained. The threshold level of the factor loading was set at > 0.50. Sampling adequacy test was performed via the Kaiser–Meyer–Olkin test. Reliability coefficients of the questionnaire were evaluated via Cronbach’s alpha to determine whether each item belonged to the assigned component scale.

Finally, we calculated each regression coefficient between the QWLQ for JCP obtained by exploratory factor analysis, QWL scale (15 items) developed by Lee [7], and QWL questionnaire (three items) to confirm whether the QWLQ for JCP explained the QWL. Validity was considered acceptable if the regression coefficient between the QWLQ for JCP and QWL scale (15 items) [7] was significantly positive.

Statistical analyses were performed using IBM SPSS version 28 and Microsoft Excel 2022. Statistical significance was set at *P* < 0.05.

## Results

### Pilot study

A total of 124 community pharmacists completed the questionnaires. Table 1 lists the respondents’ attributes. The response rate for the questionnaire was 0.70.

**Table 1:** Respondents’ attributes in the pilot study

Gender	Male	Female	Other	
	32	89	3	
Age	20 s	30 s	40 s	50 s or older
	37	57	23	7

In total, 43 of the 63 items were drawn from the question pool for the preliminary questionnaire. Furthermore, after piloting, all were retained in the pre-final version of the QWLQ for JCP.

A principal component analysis (PCA) with Promax rotation was conducted to reveal the QWL components in the pilot study. These QWL components, in reference to previous studies, were: “relationships with colleagues (Cronbach’s α = 0.850),” “opportunity of growth (α = 0.898),” “influence of work on mind and body (α = 0.813),” “meaning of existence in the workplace (α = 0.679),” “salary and benefits (α = 0.680),” and “autonomy at the workplace(α = 0.690).” These six components were finalized based on variables with a loading of at least 0.40 on a single factor and eigenvalues greater than 1. These components account for 55.540% of the total variance.

### Field study

In total, 20 companies participated. Of the 2027 community pharmacists, 1966 answered all the questions (97%). Data from 61 participants who did not provide complete information were excluded, which left 1966 participants whose data were analyzed. Tables 2 and 3 list participants’ and participating companies’ information, respectively and. The average response rate was 18.7%. Table 3 lists the response rates of each company.

**Table 2:** Respondents’ attributes in the field study

Gender	Male	Female	Other		
	721	1241	4		
Age	20 s	30 s	40 s	50 s	60 s or older
	486	838	385	182	75
Employment status: Full-time and Part-time	Full-time	Part-time			
	1710	256			
Number of employees during business hours	Number of pharmacists (Average)	Number of non-pharmacists (Average)			
	4.24	3.13			
	(Median)	(Median)			
	3	2			
	(Mode)	(Mode)			
	3	2			
Duration of service in a company	Less than 1 h	1–3 h	3–5 h	5–10 h	10–15 h
	139	358	285	545	338
	15–20 h	20 or more hr			
	177	124			
Years of pharmacist experience	Less than 1	1–3	3–5	5–10	10–15
	92	240	197	499	371
	15–20	20 or more			
	248	319			
Working hours per week	Less than 12	12–24	24–32	32–40	40 or more
	36	52	149	385	1344
Average number of prescriptions filled per a day	Less than 40	40–80	80–120	120–160	160–200
	257	698	544	193	132
	200–300	300–500	500 or more		
	99	22	21

**Table 3:** Basic information on the participating companies

Company (20 participating companies)	Number of samples (*n* = 1966)	Number of pharmacies in each company (company size)	Response rate (%)
A	1	100	100
B	9	22	10.0
C	6	2	60
D	103	60	83.1
E	157	145	19.5
F	160	1100	53.3
G	388	800	19.2
H	153	900	51.0
I	263	430	20.2
J	4	150	0.70
K	105	92	52.5
L	133	760	54.3
M	68	100	65.3
N	55	235	27.5
O	98	600	4.73
P	210	400	10.1
Q	43	22	66.2
R	9	4	100
S	1	300	100

### Item analysis

The item analysis revealed that ceiling and floor effects were not confirmed in the questionnaire. Furthermore, six items for which more than 50% of the respondents selected the same option were excluded from the questionnaire. In addition, two items with a correlation coefficient of 0.7 or more were extracted, and the item with the lowest variance was excluded from the exploratory factor analysis. Consequently, two items were excluded.

Finally, the 35-item pre-final version of the QWLQ for JCP was analyzed via an exploratory factor analysis.

### Exploratory factor analysis

An exploratory factor analysis was conducted to reduce the number of components via principal component analysis (PCA) with Promax rotation. Tables 4 and 5 present a summary of the PCA results. Based on the principal component analysis and in reference to previous studies, five predominant QWL components with eigenvalues of greater than 1 were selected: “influence of work on mind and body,” “relationships with colleagues,” “relationship with the boss,” “meaning of existence in the workplace,” and “pride in work.” The QWLQ for JCP had 18 items with five components and was finalized based on those variables with a loading of at least 0.50 on a single factor. This was since factor loadings of 0.50 or greater were “practically significant” for a sample size of 100 [28]. Cronbach’s alpha, a measure of internal consistency and reliability, was 0.585 for “pride in work” and 0.854 for “influence of work on mind and body.” These components accounted for 54.286% of the total variance.

**Table 4:** Summary of PCA I

Factors	Measurable values	Weights	Eigenvalues	Dispersion	Accumulated	Cronbach’s alpha
Influence of work on mind and body	I think the amount of work normally required is appropriate	0.806	6.212	34.509	34.509	0.854
	I am satisfied with the number of hours I work per week	0.789				
	I am satisfied with my work-life balance	0.78				
	I am satisfied with the working conditions	0.775				
	I think the number of employees in my pharmacy is adequate	0.609				
	My company offers flexible working conditions that fit into my personal life	0.608				
	I feel work-related stress. (Reverse item)	0.568				
Relationships with	I think my relationship with my colleagues is good	0.901	2.187	12.15	46.659	0.839
	My colleagues teach me about work properly	0.766				
	I believe that there is good communication among all staff members	0.734				
	When I consult with my colleagues, they give me useful information	0.516				
Relationship with boss	The boss respects the opinions of his subordinates	0.891	1.534	8.52	55.18	0.83
	My bosses are fair to their employees	0.756				
	I have a boss that I respect	0.718				
Meaning of existence in the workplace	I have a clear understanding of my role in the workplace	0.726	1.104	6.132	61.312	0.631
	I can handle the work that I should be in charge of (patient care, etc.) by myself	0.622				
Pride in work	I think that pharmacists are necessary for Japanese medical care	0.703	1.021	5.675	66.987	0.585
	Working as a pharmacist makes me want to work hard at self-improvement	0.567

**Table 5:** Summary of PCAII

Total Variance Explained						
Component	Initial Eigenvalues			Extraction Sums of Squared Loadings		
	Total	% of Variance	Cumulative %	Total	% of Variance	Cumulative %
1	6.212	34.509	34.509	5.784	32.132	32.132
2	2.187	12.15	46.659	1.763	9.796	41.928
3	1.534	8.52	55.18	1.086	6.035	47.963
4	1.104	6.132	61.312	0.642	3.569	51.532
5	1.021	5.675	66.987	0.496	2.754	54.286
6	0.729	4.048	71.035			
7	0.649	3.603	74.638			
8	0.603	3.352	77.99			
9	0.556	3.091	81.081			
10	0.529	2.936	84.018			
11	0.497	2.761	86.779			
12	0.437	2.426	89.204			
13	0.385	2.137	91.341			
14	0.355	1.97	93.311			
15	0.328	1.82	95.131			
16	0.323	1.796	96.926			
17	0.284	1.579	98.505			
18	0.269	1.495	100			

The Kaiser–Meyer–Olkin test measure, a sampling adequacy test, was 0.893. Bartlett’s Test of Sphericity (14996.935 df. 153, Sig. 0.00) showed that the values were significant, which indicated that non-zero correlations existed at a significance level of 0.00 (Table 6).

**Table 6:** Results of the KMO and Bartlett’s test

Kaiser–Meyer–Olkin (KMO) test		
Kaiser–Meyer–Olkin (KMO) value		0.893
Barlett's Test of Sphericity	Approximate chi-square	14,996.935
	Degree of freedom	153
	Significance probability	0.000

In addition, we examined whether scores from the QWLQ for JCP explained overall job satisfaction, intention to continue working, and life satisfaction outside work. A simple regression analysis was performed. The estimates of overall scores for job satisfaction, intention to continue working, and life satisfaction outside work were all significant for the QWL scale (15 items) [7] (Table 7). Therefore, the construct validity of the QWLQ for JCP regarding external criteria was confirmed.

**Table 7:** Results of the verification of predictive validity of the QWL via a regression analysis

Dependent variable	Independent variable	Standardized regression coefficient β	*R*-squared value
QWL score, Lee (2003)	QWL score for JCP	0.804	0.64
Overall job satisfaction	QWL score for JCP	0.669	0.447
Intention to continue working	QWL score for JCP	0.628	0.394
Satisfaction with life outside work	QWL score for JCP	0.406	0.164

## Discussion

To develop a preliminary version of the QWLQ for JCP, we used the QWL components reported by Ono [13], who reviewed QWL studies in the United States, for several reasons. First, Ono’s study [13] was comprehensively organized and used to explore QWL components among Japanese community pharmacists. Second, we considered that these QWL components accounted for almost all QWL components relevant to healthcare workers. Hence, we considered the developed questionnaire as an appropriate preliminary version of the QWLQ for JCP.

In the pilot and field studies, an item analysis was conducted to develop a simple QWL scale of sufficient quality for Japanese community pharmacists. Items with ceiling or floor effects and those for which more than 50% of the respondents selected the same option were excluded. Items with small variations in responses were considered unsuitable in measurement scales. In addition, if there were items with high correlation coefficients and similar content, one item with lower variance was excluded from the preliminary version.

Part of this study was an exploratory factor analysis that used a principal component analysis with Promax rotation to reduce the number of items and determine the predominant QWL components in Japanese community pharmacists. An appropriate dataset was indicated if item loading was greater than 0.5 [29]. In this study, item loadings ranged from 0.516–0.901. Hence, we considered that items for the QWLQ for JCP with a loading of 0.50 were appropriate. The Kaiser–Meyer–Olkin test was performed to assess the adequacy of the sample for the exploratory factor analysis. It measured the adequacy of the sample for the individual model variables, as well as the entire model. According to Kaiser and Rice [30], if a sample was adequate, the KMO value should be greater than 0.6. Hence, we considered a value of 0.893 value as acceptable. Bartlett’s Test of Sphericity was conducted on the collected data and demonstrated values of an approximate chi-square of 14,996.935, with 153 degrees of freedom, and a significance level of 0.000, which indicated that the values were within an acceptable range. This implied that non-zero correlations existed at a significance level of 0.000, which indicated that the data were non-spherical and sufficient for conducting a factor analysis.

Cronbach’s alpha ranged from 0.585 for “pride in work” to 0.854 for “influence of work on mind and body.” In particular, Cronbach's alpha coefficients for factors 4 and 5 were 0.631 and 0.585, respectively, which indicated low reliability. However, according to Wakako [31], pharmacists in Japan did not feel "motivated" by their work, which implied they also might not experience “meaning of existence in the workplace” or “pride in work.” Hence, we decided to leave these items in the QWLQ for JCP as these factors were important to measure Japanese pharmacists’ QWL.

The QWL score for the QWLQ for JCP significantly explained “willingness to continue working,” “life satisfaction outside work,” and “overall job satisfaction,” according to simple regression analysis. This indicated that QWL affected overall job satisfaction and also non-work life satisfaction. We believe that these results supported the general interpretation of QWL. In addition, since the QWL score of the QWLQ for JCP significantly explained the QWL scale developed by Lee [7], we concluded that the QWLQ for JCP was an appropriate QWL scale.

In addition, in the pilot study, the distribution of response attributes was biased toward young people. Therefore, we conducted a factor analysis with data from 126 people in the pilot study to examine the influence of age distribution. The factors of QWL in the pilot study were generally similar to those in the field study, which suggested that the influence of age distribution was low. Japanese pharmacists’ age distribution was 20,334, 47,465, 44,436, 37,234, and 37,513 in their 20 s, 30 s, 40 s, 50 s, and 60 s or older, respectively [32]. The age distribution in this study was biased toward young people. However, considering that pharmacists in their 40 s and 50 s were often promoted to managerial positions outside the pharmacy, such as area managers, the age distribution did not have a large effect on the QWLQ for JCP.

This survey was limited to pharmacists who primarily belonged to dispensing pharmacies operated by large companies. Responses of pharmacists who belonged to small- and medium-sized community pharmacies may not have been fully reflected in the QWLQ for JCP. Therefore, examining the scale’s external validity remains a task for future research.

The QWLQ for JCP may be useful for measuring community pharmacists’ QWL and managing their motivation to provide pharmaceutical services. We believe that this study is necessary for holistically developing community pharmacists in Japan.

## Conclusions

This study identified five QWL components based on an exploratory factor analysis: “influence of work on mind and body,” “relationship with colleagues,” “relationship with boss,” “meaning of existence in the workplace,” and “pride in work.” Our results provide reasonable support for the validity and reliability of the developed scale, which can be used to measure QWL in community pharmacists. Hence, the QWLQ for JCP is a useful instrument. However, additional data are required to ensure its internal and external validities.

### Supplementary Information

**Supplementary Material 1. **

## Data Availability

Datasets supporting the conclusions of this study are included in the article and additional files.

## Abbreviations

QWL: Quality of Work (or Working) Life
QWLQ: Quality of Work (or Working) Life Questionnaire
JCP: Japanese Community Pharmacists
QWLQ for JCP: QWL questionnaire for Japanese Community Pharmacists
ERG: Existence, Relatedness, and Growth
MHLW: The Ministry of Health, Labour and Welfare
APhA: American Pharmaceutical Association
PCA: Principal Component Analysis
KMO: Kaiser–Meyer–Olkin

## References

[1] Walton RE, Davis LE, Cherns AB (1975). Criteria for quality of working life. The quality of working life.

[2] Morton HC (1977). A look at factors affecting the quality of work life. Mon Lab Rev.

[3] Bowditch JL, Buono AF (1982). Quality of work life assessment.

[4] Levine MF, Taylor JC, Davis LE (1984). Defining quality of work. Hum Relat.

[5] Morita M (1993). [Humanization of work today] konnichiniokeru roudou no ningenka no tenkai. Bull Fac Sociol Kansai University.

[6] Swamy DR, Nanjundeswaraswamy TS, Rashmi S (2015). Quality of work life: scale development and validation. Int J Caring Sci.

[7] Lee JW (2006). Development of a Japanese measurement scale of quality of working life for staff working in nursing facilities for the elderly. Soc Sci Jpn J.

[8] Nanjundeswaraswamy TS (2021). Nurses quality of work life: scale development and validation. J Econ Admin Sci.

[9] Zaman S, Ansari AH. Quality of work-life: scale construction and validation. J Econ Admin Sci. 2022. ahead-of-print, Issue ahead-of-print. 10.1108/JEAS-07-2021-0118.

[10] McHugh PP (1999). Pharmacists’ attitudes regarding quality of worklife. J Am Pharm Assoc (2003).

[11] Midwest Pharmacy Workforce Research Consortium (2015). National Pharmacist Workforce Survey.

[12] Schommer JC, Gaither CA, Doucette WR, Kreling DH, Mott DA (2018). Associations between work activity and work setting categories and dimensions of pharmacists’ quality of work life. Pharmacy (Basel).

[13] Ono K (1986). [Humanizing Labor: Focusing on QWL in the United States] Roudou no ningenka: beikoku no QWL wo tyuushintoshite. Asia Univ Manag Rev.

[14] Ministry of Health, Labour and Welfare. Vision of pharmacy useful for patients. Notification 1023 No. 3 of 2015 of the PFSB/GAD (Japanese). 2015. Accessed

[15] Rastegari M, Khani A, Ghalriz P, Eslamian J (2010). Evaluation of quality of working life and its association with job performance of the nurses. Iran J Nurs Midwif Res.

[16] Borhani F, Arbabisarjou A, Kianian T, Saber S (2016). Assessment of predictable productivity of nurses working in Kerman University of Medical Sciences' teaching hospitals via the dimensions of quality of work life. Glob J Health Sci.

[17] Rajamohan S, Porock D, Chang YP (2019). Understanding the relationship between staff and job satisfaction, stress, turnover, and staff outcomes in the person-centered care nursing home arena. J Nurs Scholarsh.

[18] Urbonas G, Kubilienė L (2016). Assessing the relationship between pharmacists' job satisfaction and over-the-counter counselling at community pharmacies. Int J Clin Pharm.

[19] Chui MA, Look KA, Mott DA (2014). The association of subjective workload dimensions on quality of care and pharmacist quality of work life. Res Social Adm Pharm.

[20] Kreling DH, Doucette WR, Mott DA, Gaither CA, Pedersen CA, Schommer JC (2003). Community pharmacists’ work environments: evidence from the 2004 National Pharmacist Workforce Study. J Am Pharm Assoc.

[21] Malone DC, Abarca J, Skrepnek GH, Murphy JE, Armstrong EP, Grizzle AJ (2007). Pharmacist workload and pharmacy characteristics associated with the dispensing of potentially clinically important drug-drug interactions. Med Care.

[22] Rupp MT, DeYoung M, Schondelmeyer SW (1992). Prescribing problems and pharmacist interventions in community practice. Med Care.

[23] Gidman WK, Hassell K, Day J, Payne K (2007). The impact of increasing workloads and role expansion on female community pharmacists in the United Kingdom. Res Social Adm Pharm.

[24] Eden M, Schafheutle EI, Hassell K (2009). Workload pressure among recently qualified pharmacists: an exploratory study of intentions to leave the profession. Int J Pharm Pract.

[25] Danganan JB, Velasquez ZF, Guinto KMP, Mac Ardy JG (2019). Quality of worklife of pharmacists in the Philippines: a descriptive, correlational study. J Asian Assoc Sch Pharm.

[26] da Silva TL, Pedroso B, de Francisco AC, Pilatti LA. Evaluation of quality of work life: an adaptation from the Walton’s QWL model. In: XIV International Conference on Industrial Engineering and Operations Management. United Arab Emirates; 2008;1:13–16.

[27] Shimizu T. Ganso Okkamu no kamisori. Kikan Tetugaku. Japan: Tetugaku Shobou; 1990;11:8–23 (in Japanese).

[28] Hair JF, Black WC, Babin BJ, Anderson RE, Tatham RL (2009). Multivariate data analysis.

[29] Stewart DW (1981). The application and misapplication of factor analysis in marketing research. J Mark Res.

[30] Kaiser HF, Rice J (1974). Little Jiffy, Mark IV. Educ Psychol Meas.

[31] Wakako N. [Pharmacist Future Progressive] Yakuzaishi no Miraishinkoukei. Japan: Yakuji Nippo; 2020. p. 144 (in Japanese).

[32] Ministry of Health, Labour and Welfare. Overview of 2020 physician, dentist, and pharmacist statistics. 2020 nen no ishi-shikaishi-yakuzaishiz toukei no gaiyo (in Japanese). 2020. https://www.mhlw.go.jp/toukei/saikin/hw/ishi/20/dl/R02_kekka-3.pdf. Accessed.

