# Supplementary material for: Development of a quality of work life scale for Japanese community pharmacists: a questionnaire survey mostly in large companies
Source: J Pharm Health Care Sci. 2024 Mar 11;10:16. doi: 10.1186/s40780-024-00335-z (PMC10926542; doi:10.1186/s40780-024-00335-z)
Supplement: Supplementary file 1 — Supplementary Material 1. [file 40780_2024_335_MOESM1_ESM.zip › The questionnaire No.1 .pdf]

QWL質問票

...

こんにちは、裕太。このフォームを送信すると、所有者に名前とメールアドレスが表示されます。

質問票について

質問は、薬剤師QWL尺度質問票(43項目)、基本属性質問票(15項目)、主観的パフォーマンス評価質問票(7項目)、既存QWL尺度(15項目)、QWL尺度(定義)質問票(3項目)の全5セクションで構成されており、全83項目です。各セクションの指示にしたがって回答してください。(感覚的に、ご回答してください。)

次へ

このコンテンツはフォームの所有者が作成したものです。送信したデータはフォームの所有者に送信されます。
Microsoft は、このフォームの所有者を含むお客様のプライバシーやセキュリティの取り扱いに関して一切の責任を負いません。パスワードを記載しないでください。

Powered by Microsoft Forms | プライバシーと Cookie | 利用規約
