# Supplementary material for: Development of a quality of work life scale for Japanese community pharmacists: a questionnaire survey mostly in large companies
Source: J Pharm Health Care Sci. 2024 Mar 11;10:16. doi: 10.1186/s40780-024-00335-z (PMC10926542; doi:10.1186/s40780-024-00335-z)
Supplement: Supplementary file 1 — Supplementary Material 1. [file 40780_2024_335_MOESM1_ESM.zip › The questionnaire No.2.pdf]

QWL質問票 ...

倫理的配慮について

　本研究にあたっては、貴社及びご回答いただいた方々に不利益が生じないよう、当研究室の責任において個人情報の保護、プライバシーの尊重に努力し最大限の注意を払います。個人特定の可能性のある回答は、研究実施者によって匿名化された後、研究に用いられます。また、研究と無関係の第三者に匿名化した個人情報を提供することはありません。
　この研究にご協力いただくかどうかは、あなたの自由意思に委ねられています。本研究では、アンケート回答を送信していただくことで、「同意」とさせていただきます。なお、研究にご協力いただけないことで、あなたの不利益に繋がることは一切ありません。もし、同意を撤回し、参加を拒否したい場合は、そのまま回答を中止してください。回答を送信しない限り、回答内容の記録は残りません。
　ただし、送信してしまった場合、調査の回答が完全に匿名化され、個人の特定が不可能である場合、同意の撤回を断らせていただきます。

戻る　　次へ

このコンテンツはフォームの所有者が作成したものです。送信したデータはフォームの所有者に送信されます。Microsoft は、このフォームの所有者を含むお客様のプライバシーやセキュリティの取り扱いに関して一切の責任を負いません。パスワードを記載しないでください。

Powered by Microsoft Forms | プライバシーと Cookie | 利用規約
