# Supplementary material for: Development of a quality of work life scale for Japanese community pharmacists: a questionnaire survey mostly in large companies
Source: J Pharm Health Care Sci. 2024 Mar 11;10:16. doi: 10.1186/s40780-024-00335-z (PMC10926542; doi:10.1186/s40780-024-00335-z)
Supplement: Supplementary file 1 — Supplementary Material 1. [file 40780_2024_335_MOESM1_ESM.zip › The questionnaire No.3.pdf]

QWL質問票

* 必須

薬剤師QWL尺度質問票

質問は全部で43項目です。
「全く当てはまらない」に1、「ほとんど当てはまらない」に2、「あまり当てはまらない」に3、「やや当てはまる」に4、「かなり当てはまる」に5、「非常に当てはまる」に6でお答えください。

1. 上司は、職員に対して公平に関わっている。
*

- 1 全く当てはまらない
- 2 ほとんど当てはまらない
- 3 あまり当てはまらない
- 4 やや当てはまる
- 5 かなり当てはまる
- 6 非常に当てはまる

2. 尊敬できる上司がいる。
*

- 1 全く当てはまらない
- 2 ほとんど当てはまらない

- 3　あまり当てはまらない
- 4　やや当てはまる
- 5　かなり当てはまる
- 6　非常に当てはまる

3. 上司は、部下の意見を尊重してくれている。
*

- 1　全く当てはまらない
- 2　ほとんど当てはまらない
- 3　あまり当てはまらない
- 4　やや当てはまる
- 5　かなり当てはまる
- 6　非常に当てはまる

4. 自分の仕事内容は、多岐にわたる。
*

- 1　全く当てはまらない
- 2　ほとんど当てはまらない
- 3　あまり当てはまらない
- 4　やや当てはまる
- 5　かなり当てはまる
- 6　非常に当てはまる

5. 医療従事者としての薬剤師の職能に満足している。
*

- 1　全く当てはまらない
- 2　ほとんど当てはまらない
- 3　あまり当てはまらない
- 4　やや当てはまる
- 5　かなり当てはまる
- 6　非常に当てはまる

6. 認定薬剤師などの資格取得に挑戦できる環境である。
*

- 1　全く当てはまらない
- 2　ほとんど当てはまらない
- 3　あまり当てはまらない
- 4　やや当てはまる
- 5　かなり当てはまる
- 6　非常に当てはまる

7. 職場は、自分の考えを述べられる環境である。
*

- 1　全く当てはまらない
- 2　ほとんど当てはまらない

- 3　あまり当てはまらない
- 4　やや当てはまる
- 5　かなり当てはまる
- 6　非常に当てはまる

8. 今の職場は、薬剤師として働く上で勉強になる。
*

- 1　全く当てはまらない
- 2　ほとんど当てはまらない
- 3　あまり当てはまらない
- 4　やや当てはまる
- 5　かなり当てはまる
- 6　非常に当てはまる

9. 今の職場は、薬剤師として成長できる機会がある。
*

- 1　全く当てはまらない
- 2　ほとんど当てはまらない
- 3　あまり当てはまらない
- 4　やや当てはまる
- 5　かなり当てはまる
- 6　非常に当てはまる

10. 自分は昇進できる可能性があると思う。 *

- 1　全く当てはまらない
- 2　ほとんど当てはまらない
- 3　あまり当てはまらない
- 4　やや当てはまる
- 5　かなり当てはまる
- 6　非常に当てはまる

11. 自分が薬剤師であることを誇りに思う。 *

- 1　全く当てはまらない
- 2　ほとんど当てはまらない
- 3　あまり当てはまらない
- 4　やや当てはまる
- 5　かなり当てはまる
- 6　非常に当てはまる

12. 仕事と仕事以外の生活のバランスに、満足している。 *

- 1　全く当てはまらない
- 2　ほとんど当てはまらない
- 3　あまり当てはまらない
- 4　やや当てはまる

4 やや当てはまる

5 かなり当てはまる

6 非常に当てはまる

13. 仕事が自分の健康に影響することがある。(逆転項目)
*

1 全く当てはまらない

2 ほとんど当てはまらない

3 あまり当てはまらない

4 やや当てはまる

5 かなり当てはまる

6 非常に当てはまる

14. 自分の存在は、所属する薬局店舗に必要だと思う。
*

1 全く当てはまらない

2 ほとんど当てはまらない

3 あまり当てはまらない

4 やや当てはまる

5 かなり当てはまる

6 非常に当てはまる

15. 会社からの補償（福利厚生、健康診断の実施等）に満足している。

*

- 1　全く当てはまらない
- 2　ほとんど当てはまらない
- 3　あまり当てはまらない
- 4　やや当てはまる
- 5　かなり当てはまる
- 6　非常に当てはまる

16. 自分の給料は、自分が会社にする貢献に見合っている。
*

- 1　全く当てはまらない
- 2　ほとんど当てはまらない
- 3　あまり当てはまらない
- 4　やや当てはまる
- 5　かなり当てはまる
- 6　非常に当てはまる

17. 週あたりの勤務時間には、満足している。
*

- 1　全く当てはまらない
- 2　ほとんど当てはまらない
- 3　あまり当てはまらない
- 4　やや当てはまる

[illegible]

5 かなり当てはまる

6 非常に当てはまる

18. 普段求められている業務量は適切であると思う。
*

1 全く当てはまらない

2 ほとんど当てはまらない

3 あまり当てはまらない

4 やや当てはまる

5 かなり当てはまる

6 非常に当てはまる

19. 職場のみんなは、会社から公平に評価されている。
*

1 全く当てはまらない

2 ほとんど当てはまらない

3 あまり当てはまらない

4 やや当てはまる

5 かなり当てはまる

6 非常に当てはまる

20. 職場における宗教、性別、人種等の多様性は尊重されている。

*

- 1　全く当てはまらない
- 2　ほとんど当てはまらない
- 3　あまり当てはまらない
- 4　やや当てはまる
- 5　かなり当てはまる
- 6　非常に当てはまる

21. 給料以外の補償（福利厚生等）は、職員に公平に与えられている。
*

- 1　全く当てはまらない
- 2　ほとんど当てはまらない
- 3　あまり当てはまらない
- 4　やや当てはまる
- 5　かなり当てはまる
- 6　非常に当てはまる

22. 同僚と相談した際は、ためになる情報を教えてくれる。
*

- 1　全く当てはまらない
- 2　ほとんど当てはまらない
- 3　あまり当てはまらない
- 4　やや当てはまる

- 5　かなり当てはまる
- 6　非常に当てはまる

23. 自分が担当すべき業務（患者対応等）は、一人で対応できる。
*

- 1　全く当てはまらない
- 2　ほとんど当てはまらない
- 3　あまり当てはまらない
- 4　やや当てはまる
- 5　かなり当てはまる
- 6　非常に当てはまる

24. 職場での自分の役割をはっきり認識している。
*

- 1　全く当てはまらない
- 2　ほとんど当てはまらない
- 3　あまり当てはまらない
- 4　やや当てはまる
- 5　かなり当てはまる
- 6　非常に当てはまる

25. 職場での表現の自由さ（自分の意見を言える等）に満足している。
*

*

- 1　全く当てはまらない
- 2　ほとんど当てはまらない
- 3　あまり当てはまらない
- 4　やや当てはまる
- 5　かなり当てはまる
- 6　非常に当てはまる

26. 薬局の業務体制を変更または改善する際、意見交換に参加できる。 *

- 1　全く当てはまらない
- 2　ほとんど当てはまらない
- 3　あまり当てはまらない
- 4　やや当てはまる
- 5　かなり当てはまる
- 6　非常に当てはまる

27. 自分の仕事内容について、同僚に意見することができる。 *

- 1　全く当てはまらない
- 2　ほとんど当てはまらない
- 3　あまり当てはまらない
- 4　やや当てはまる

◯ 5　かなり当てはまる

◯ 6　非常に当てはまる

28. 職場の安全対策システムは十分に施されている。
*

◯ 1　全く当てはまらない

◯ 2　ほとんど当てはまらない

◯ 3　あまり当てはまらない

◯ 4　やや当てはまる

◯ 5　かなり当てはまる

◯ 6　非常に当てはまる

29. 自分の薬局の従業員の人数は適切である。
*

◯ 1　全く当てはまらない

◯ 2　ほとんど当てはまらない

◯ 3　あまり当てはまらない

◯ 4　やや当てはまる

◯ 5　かなり当てはまる

◯ 6　非常に当てはまる

30. 薬局にある、業務上必要な器具（分包機やはかり等）について満足している。
*

- 1　全く当てはまらない
- 2　ほとんど当てはまらない
- 3　あまり当てはまらない
- 4　やや当てはまる
- 5　かなり当てはまる
- 6　非常に当てはまる

31. 自分と同僚の関係は良いと思う。
*

- 1　全く当てはまらない
- 2　ほとんど当てはまらない
- 3　あまり当てはまらない
- 4　やや当てはまる
- 5　かなり当てはまる
- 6　非常に当てはまる

32. 職員全体で、十分なコミュニケーションを取れていると思う
*

- 1　全く当てはまらない
- 2　ほとんど当てはまらない
- 3　あまり当てはまらない
- 4　やや当てはまる

○ 5　かなり当てはまる

○ 6　非常に当てはまる

33. 同僚は、仕事について適切に教えてくれる。
*

○ 1　全く当てはまらない

○ 2　ほとんど当てはまらない

○ 3　あまり当てはまらない

○ 4　やや当てはまる

○ 5　かなり当てはまる

○ 6　非常に当てはまる

34. 薬剤師として働いていると、自己研鑽に励みたくなる。
*

○ 1　全く当てはまらない

○ 2　ほとんど当てはまらない

○ 3　あまり当てはまらない

○ 4　やや当てはまる

○ 5　かなり当てはまる

○ 6　非常に当てはまる

35. 薬剤師は、日本の医療に必要な存在だと思う。
*

1　全く当てはまらない

2　ほとんど当てはまらない

3　あまり当てはまらない

4　やや当てはまる

5　かなり当てはまる

6　非常に当てはまる

36. 現在の職場は、学ぶ機会が十分にある。
*

1　全く当てはまらない

2　ほとんど当てはまらない

3　あまり当てはまらない

4　やや当てはまる

5　かなり当てはまる

6　非常に当てはまる

37. 今まで学んだ薬学的な知識は、役に立っている。
*

1　全く当てはまらない

2　ほとんど当てはまらない

3　あまり当てはまらない

4　やや当てはまる

- 5　かなり当てはまる
- 6　非常に当てはまる

38. 業務上のストレスを感じる。(逆転項目)
*

- 1　全く当てはまらない
- 2　ほとんど当てはまらない
- 3　あまり当てはまらない
- 4　やや当てはまる
- 5　かなり当てはまる
- 6　非常に当てはまる

39. 薬剤師の仕事は達成感を感じることができる。
*

- 1　全く当てはまらない
- 2　ほとんど当てはまらない
- 3　あまり当てはまらない
- 4　やや当てはまる
- 5　かなり当てはまる
- 6　非常に当てはまる

40. 薬剤師業務で感じるプレッシャーは、妥当だと思う。
*

- 1　全く当てはまらない
- 2　ほとんど当てはまらない
- 3　あまり当てはまらない
- 4　やや当てはまる
- 5　かなり当てはまる
- 6　非常に当てはまる

41. 私の会社は、私生活に調和するように柔軟な労働条件を提示してくれる。 *

- 1　全く当てはまらない
- 2　ほとんど当てはまらない
- 3　あまり当てはまらない
- 4　やや当てはまる
- 5　かなり当てはまる
- 6　非常に当てはまる

42. 業務時間外ではストレスを感じない。 *

- 1　全く当てはまらない
- 2　ほとんど当てはまらない
- 3　あまり当てはまらない
- 4　やや当てはまる

- 5　かなり当てはまる
- 6　非常に当てはまる

43. 労働条件に満足している。
*

- 1　全く当てはまらない
- 2　ほとんど当てはまらない
- 3　あまり当てはまらない
- 4　やや当てはまる
- 5　かなり当てはまる
- 6　非常に当てはまる

戻る　　次へ

このコンテンツはフォームの所有者が作成したものです。送信したデータはフォームの所有者に送信されます。Microsoft は、このフォームの所有者を含むお客様のプライバシーやセキュリティの取り扱いに関して一切の責任を負いません。パスワードを記載しないでください。

Powered by Microsoft Forms | プライバシーと Cookie | 利用規約
