# Supplementary material for: Development of a quality of work life scale for Japanese community pharmacists: a questionnaire survey mostly in large companies
Source: J Pharm Health Care Sci. 2024 Mar 11;10:16. doi: 10.1186/s40780-024-00335-z (PMC10926542; doi:10.1186/s40780-024-00335-z)
Supplement: Supplementary file 1 — Supplementary Material 1. [file 40780_2024_335_MOESM1_ESM.zip › The questionnaire No.4.pdf]

QWL質問票

* 必須

基本属性質問票

質問は全部で15問です。
回答は選択式および記述式です。各質問の指示にしたがって回答してください。

44. 性別を選択してください。 *

- 1　男性
- 2　女性
- 3　その他

45. 年齢を下記からお選びください。 *

- 1　：20代
- 2　：30代
- 3　：40代
- 4　：50代
- 5　：60代
- 6　：70代以上

46. 雇用形態を選択してください。 *

- 1　正社員
- 2　契約社員・パート社員

47. 現在勤務している会社の勤続年数を選択してください。 *

- 1　満1年未満
- 2　満1年以上-満3年未満
- 3　満3年以上-満5年未満
- 4　満5年以上-満10年未満
- 5　満10年以上-満15年未満
- 6　満15年以上-満20年未満
- 7　満20年以上

48. 働いている薬局は、門前薬局ですか？(主観的な回答で構いません) *

- 1　はい
- 2　いいえ

49. 薬剤師経験年数(病院・薬局等で薬剤師業務に従事した年数)を選択してください。 *

- 1　満1年未満
- 2　満1年以上-満3年未満
- [illegible]

- 3 満3年以上-満5年未満
- 4 満5年以上-満10年未満
- 5 満10年以上-満15年未満
- 6 満15年以上-満20年未満
- 7 満20年以上

50. 週あたりの勤務時間(h)を選択してください。 *

- 1 ：12h未満
- 2 ：12h以上-24h未満
- 3 ：24h以上-32h未満
- 4 ：32h以上-40h未満
- 5 ：40h以上

51. あなたは、薬局責任者(薬局長または管理薬剤師)ですか？ *

- 1 はい
- 2 いいえ

52. あなたは普段、かかりつけ薬剤師指導料もしくは、かかりつけ薬剤師包括管理料を算定していますか？ *

- 1 はい
- 2 いいえ

53. 所属薬局の平均応需処方箋枚数/日をお答えください。 *

- 1　：40未満
- 2　：40以上-80未満
- 3　：80以上-120未満
- 4　：120以上-160未満
- 5　：160以上-200未満
- 6　：200以上-300未満
- 7　：300以上-500未満
- 8　：500以上

54. 自分が所属する会社(ホールディングス、グループ)を選択してください。
下記のリストに該当する選択肢がない場合は、その他を選択し、所属会社をご入力ください。
*

- 1　I&H
- 2　アイセイ薬局
- 3　アインホールディングス
- 4　アモール
- 5　いずみ調剤
- 6　共栄堂
- 7　クオール
- 8　クラフト
- 9　総合メディカル

- 10　タカラ薬局
- 11　たんぽぽ薬局
- 12　中央薬品
- 13　ドラッグスソウ
- 14　永冨調剤
- 15　ノムラ薬局
- 16　ピノキオ薬局
- 17　ファーマシィ
- 18　ファーマライズホールディングス
- 19　フロンティア
- 20　ミアヘルサ
- 21　メディカルシステムネットワーク
- 22　メディカル一光
- 23　薬樹
- 24　レディ薬局
- 25　わかば
- その他

55. 直近3カ月間でのご自身の在宅実績はありますか？ *

- 1　はい
- 2　いいえ

56. 営業時の薬剤師人数を整数で答えてください。※単位はつけないでください。 *

回答を入力してください

57. 営業時の薬剤師でない人の人数を整数で答えてください。※単位はつけないでください。 *

回答を入力してください

58. 所属している薬局の都道府県と市区町村を教えてください。(例：東京都板橋区、千葉県千葉市など)
*

回答を入力してください

59. 経験薬局店舗数を教えてください(応援・サポート等の臨時的な勤務経験は除く) 。
※整数でお答えください。単位はつけないでください。
*

回答を入力してください

戻る　次へ

このコンテンツはフォームの所有者が作成したものです。送信したデータはフォームの所有者に送信されます。
Microsoft は、このフォームの所有者を含むお客様のプライバシーやセキュリティの取り扱いに関して一切の責任を負いません。パスワードを記載しないでください。

Powered by Microsoft Forms | プライバシーと Cookie | 利用規約
