# Supplementary material for: Development of a quality of work life scale for Japanese community pharmacists: a questionnaire survey mostly in large companies
Source: J Pharm Health Care Sci. 2024 Mar 11;10:16. doi: 10.1186/s40780-024-00335-z (PMC10926542; doi:10.1186/s40780-024-00335-z)
Supplement: Supplementary file 1 — Supplementary Material 1. [file 40780_2024_335_MOESM1_ESM.zip › The questionnaire No.5.pdf]

QWL質問票

* 必須

主観的パフォーマンス評価質問票

質問は全部で7項目です。
「全く当てはまらない」に1、「ほとんど当てはまらない」に2、「あまり当てはまらない」に3、「やや当てはまる」に4、「かなり当てはまる」に5、「非常に当てはまる」に6でお答えください。

60. 服薬指導の際、処方解析ができている。 *

- 1 全く当てはまらない
- 2 ほとんど当てはまらない
- 3 あまり当てはまらない
- 4 やや当てはまる
- 5 かなり当てはまる
- 6 非常に当てはまる

61. 服薬指導の際は、薬物治療のメリット、デメリットを理解させることができている。 *

- 1 全く当てはまらない
- 2 ほとんど当てはまらない
- 3 あまり当てはまらない

- 3 あまり当てはまらない
- 4 やや当てはまる
- 5 かなり当てはまる
- 6 非常に当てはまる

62. 患者の治療に貢献できている。 *

- 1 全く当てはまらない
- 2 ほとんど当てはまらない
- 3 あまり当てはまらない
- 4 やや当てはまる
- 5 かなり当てはまる
- 6 非常に当てはまる

63. 医師に処方提案ができる。 *

- 1 全く当てはまらない
- 2 ほとんど当てはまらない
- 3 あまり当てはまらない
- 4 やや当てはまる
- 5 かなり当てはまる
- 6 非常に当てはまる

64. 患者の経過等に関して、医師に情報提供ができる。
*

- 1　全く当てはまらない
- 2　ほとんど当てはまらない
- 3　あまり当てはまらない
- 4　やや当てはまる
- 5　かなり当てはまる
- 6　非常に当てはまる

65. 服用期間中、患者の状況を把握できている。
*

- 1　全く当てはまらない
- 2　ほとんど当てはまらない
- 3　あまり当てはまらない
- 4　やや当てはまる
- 5　かなり当てはまる
- 6　非常に当てはまる

66. 服用期間中、必要に応じて、服薬フォローアップができている。
*

- 1　全く当てはまらない
- 2　ほとんど当てはまらない
- 3　あまり当てはまらない

[illegible]

4　やや当てはまる

5　かなり当てはまる

6　非常に当てはまる

戻る　次へ

このコンテンツはフォームの所有者が作成したものです。送信したデータはフォームの所有者に送信されます。
Microsoft は、このフォームの所有者を含むお客様のプライバシーやセキュリティの取り扱いに関して一切の責任を負いません。パスワードを記載しないでください。

Powered by Microsoft Forms | プライバシーと Cookie | 利用規約
