# Supplementary material for: Development of a quality of work life scale for Japanese community pharmacists: a questionnaire survey mostly in large companies
Source: J Pharm Health Care Sci. 2024 Mar 11;10:16. doi: 10.1186/s40780-024-00335-z (PMC10926542; doi:10.1186/s40780-024-00335-z)
Supplement: Supplementary file 1 — Supplementary Material 1. [file 40780_2024_335_MOESM1_ESM.zip › The questionnaire No.6.pdf]

QWL質問票

* 必須

## 既存QWL尺度

質問は全部で15項目です。
「全く当てはまらない」に1、「ほとんど当てはまらない」に2、「あまり当てはまらない」に3、「やや当てはまる」に4、「かなり当てはまる」に5、「非常に当てはまる」に6でお答えください。

67. 給料は私の年齢や業務内容に見合っていると思う。 *

- 1 全く当てはまらない
- 2 ほとんど当てはまらない
- 3 あまり当てはまらない
- 4 やや当てはまる
- 5 かなり当てはまる
- 6 非常に当てはまる

68. 仕事の成果と給料はつりあっていると思う。 *

- 1 全く当てはまらない
- 2 ほとんど当てはまらない

- 3 あまり当てはまらない
- 4 やや当てはまる
- 5 かなり当てはまる
- 6 非常に当てはまる

69. 給料は同僚と比べて妥当だと思う。
*

- 1 全く当てはまらない
- 2 ほとんど当てはまらない
- 3 あまり当てはまらない
- 4 やや当てはまる
- 5 かなり当てはまる
- 6 非常に当てはまる

70. 職場の福利厚生は適切だと思う。
*

- 1 全く当てはまらない
- 2 ほとんど当てはまらない
- 3 あまり当てはまらない
- 4 やや当てはまる
- 5 かなり当てはまる
- 6 非常に当てはまる

71. 私と上司との関係はよいと思う。
*

- 1 全く当てはまらない
- 2 ほとんど当てはまらない
- 3 あまり当てはまらない
- 4 やや当てはまる
- 5 かなり当てはまる
- 6 非常に当てはまる

72. 私と上司との間には信頼関係が成り立っている。
*

- 1 全く当てはまらない
- 2 ほとんど当てはまらない
- 3 あまり当てはまらない
- 4 やや当てはまる
- 5 かなり当てはまる
- 6 非常に当てはまる

73. 私と同僚との関係はよいと思う。
*

- 1 全く当てはまらない
- 2 ほとんど当てはまらない

- 3 あまり当てはまらない
- 4 やや当てはまる
- 5 かなり当てはまる
- 6 非常に当てはまる

## 74. 私と同僚との間には、信頼関係が成り立っている。 *

- 1 全く当てはまらない
- 2 ほとんど当てはまらない
- 3 あまり当てはまらない
- 4 やや当てはまる
- 5 かなり当てはまる
- 6 非常に当てはまる

## 75. 同僚は仕事をするうえで協力的であると思う。 *

- 1 全く当てはまらない
- 2 ほとんど当てはまらない
- 3 あまり当てはまらない
- 4 やや当てはまる
- 5 かなり当てはまる
- 6 非常に当てはまる

76. 職場の人間関係はよいと思う。
*

- 1　全く当てはまらない
- 2　ほとんど当てはまらない
- 3　あまり当てはまらない
- 4　やや当てはまる
- 5　かなり当てはまる
- 6　非常に当てはまる

77. この仕事は「やりがいがある仕事」だと思う。
*

- 1　全く当てはまらない
- 2　ほとんど当てはまらない
- 3　あまり当てはまらない
- 4　やや当てはまる
- 5　かなり当てはまる
- 6　非常に当てはまる

78. この仕事は、私の信念・信条にかなうものである。
*

- 1　全く当てはまらない
- 2　ほとんど当てはまらない
- 3　あまり当てはまらない

◯ 3 あまり当てはまらない

◯ 4 やや当てはまる

◯ 5 かなり当てはまる

◯ 6 非常に当てはまる

79. 私は、この仕事を通じて人間的に成長できていると思う。
*

◯ 1 全く当てはまらない

◯ 2 ほとんど当てはまらない

◯ 3 あまり当てはまらない

◯ 4 やや当てはまる

◯ 5 かなり当てはまる

◯ 6 非常に当てはまる

80. この仕事で自分のもつ資格が生かせると思う。
*

◯ 1 全く当てはまらない

◯ 2 ほとんど当てはまらない

◯ 3 あまり当てはまらない

◯ 4 やや当てはまる

◯ 5 かなり当てはまる

◯ 6 非常に当てはまる

81. この仕事には幅広い知識が必要であると思う。
*

- 1　全く当てはまらない
- 2　ほとんど当てはまらない
- 3　あまり当てはまらない
- 4　やや当てはまる
- 5　かなり当てはまる
- 6　非常に当てはまる

戻る　　次へ

このコンテンツはフォームの所有者が作成したものです。送信したデータはフォームの所有者に送信されます。Microsoft は、このフォームの所有者を含むお客様のプライバシーやセキュリティの取り扱いに関して一切の責任を負いません。パスワードを記載しないでください。

Powered by Microsoft Forms | プライバシーと Cookie | 利用規約
