# Supplementary material for: Development of a quality of work life scale for Japanese community pharmacists: a questionnaire survey mostly in large companies
Source: J Pharm Health Care Sci. 2024 Mar 11;10:16. doi: 10.1186/s40780-024-00335-z (PMC10926542; doi:10.1186/s40780-024-00335-z)
Supplement: Supplementary file 1 — Supplementary Material 1. [file 40780_2024_335_MOESM1_ESM.zip › The questionnaire No.7.pdf]

QWL質問票　・・・

* 必須

# QWL尺度(定義)質問票

質問は全部で3項目です。
「全く当てはまらない」に1、「ほとんど当てはまらない」に2、「あまり当てはまらない」に3、「やや当てはまる」に4、「かなり当てはまる」に5、「非常に当てはまる」に6でお答えください。

82. 全体として私は今の仕事に満足している。 *

- 1　全く当てはまらない
- 2　ほとんど当てはまらない
- 3　あまり当てはまらない
- 4　やや当てはまる
- 5　かなり当てはまる
- 6　非常に当てはまる

83. 私はこれからも今の職場で働き続けたいと思う。 *

- 1　全く当てはまらない
- 2　ほとんど当てはまらない
- 3　あまり当てはまらない
- 4　やや当てはまる

- 4　やや当てはまる
- 5　かなり当てはまる
- 6　非常に当てはまる

## 84. 仕事以外の今の生活に満足している。 *

- 1　全く当てはまらない
- 2　ほとんど当てはまらない
- 3　あまり当てはまらない
- 4　やや当てはまる
- 5　かなり当てはまる
- 6　非常に当てはまる

戻る　　送信

このコンテンツはフォームの所有者が作成したものです。送信したデータはフォームの所有者に送信されます。
Microsoft は、このフォームの所有者を含むお客様のプライバシーやセキュリティの取り扱いに関して一切の責任を負いません。パスワードを記載しないでください。

Powered by Microsoft Forms | プライバシーと Cookie | 利用規約
